# Study of a new method for the instant preparation of ice particles in ice abrasive air jet

**DOI:** 10.1038/s41598-022-22409-4

**Published:** 2022-10-19

**Authors:** Zhiping Li, Ying Zhu, Yong Liu, Chenxu Cao, Jiaojiao Wu, Fei Huang

**Affiliations:** 1 State Key Laboratory Cultivation Base for Gas Geology and Gas Control, Henan Polytechnic University, Jiaozuo, 454000 Henan China; 2 Collaborative Innovation Center of Coal Work Safety and Clean High Efficiency Utilization, Jiaozuo, 454000 Henan China; 3 China Railway Engineering Equipment Group CO., LTD, Zhengzhou, China; 4 Hunan University of Science and Technology, Xiangtan, 411100 Hunan China

**Keywords:** Energy science and technology, Engineering

## Abstract

The ice abrasive air jet is a clean surface treatment technology, which currently has limitations such as high energy consumption, uncontrollable particle size and hardness. Realizing the instant preparation and utilization of ice particles are crucial for solving the energy consumption problem. This paper based on the icing principle of heterogeneous nucleation, proposed a new method of ice making, the heat transfer mechanism of low temperature droplets was studied, and the method was proved to be feasible. Using the FLUENT solidification and melting model combined with the VOF model to calculate the freezing process of droplets, the effects of droplet particle size, initial temperature, and wall temperature on the freezing time were analyzed, and the calculation equation of the freezing time was determined, which was corrected by the icing test results. The results showed that the outside of the droplet freezes first, the liquid–solid boundary is parabolic, and the parabolic concavity increases with time and droplet size. In the freezing process, the larger the droplet size, the longer the droplet phase transition time; the higher the droplet initial temperature, the longer it took to reach the phase transition; the higher the wall temperature, the longer the ice formation time.

## Introduction

Surface treatment methods, such as paint stripping and rust removal, cause severe pollution and high energy consumption^1^. For example, sandblasting surface treatment, sand dust, and surface waste significantly pollute the operating environment, compromising the occupational safety and health of the operators. It is difficult to separate the sand dust from the waste surface mixture, and the abrasive cannot be recycled, thereby increasing the cost of pollutant and waste treatment operations^2–4^. Environmental pollution and occupational health hazards caused by the subsidiary products of chemical treatment methods are considerable, and chemical reagents reduce the physical and mechanical properties of the workpiece^5–7^. The hydraulic cleaning method uses the impact of high-pressure water jets and water sledging to destroy the rust and the adhesion of the coating to the steel plate, and the quality of rust removal is good, but the steel plate after rust removal is easy to return to rust^8^. In contrast, dry ice blasting is an efficient and environmentally friendly method for cleaning contaminated surfaces, but it requires a large amount of compressed air, which is currently one of the most expensive forms of energy used in the industry^9,10^. Therefore, the development of an environmentally friendly and efficient surface treatment is imperative. Ice abrasive air jets with high-pressure gas accelerate ice particles, whose high speed results in sufficient impact energy to process the surface of the workpiece^11,12^. The ice particles are broken and melted after impact, accelerating the settling time of solid waste and reducing the pollution of the working environment. As ice particles and solid waste do not have to be separated, engineering costs are reduced, and work efficiency is improved. The ice abrasive air jet has unique advantages in the field of surface treatment; surface cleaning by ice particle jetting was pioneered by Galecki^13^ in Poland, who prepared ice particles by breaking a 3 cm^3^ block of ice and used a high speed air stream to entrain the ice particles. The particles were drawn in, mixed with the high-speed airflow, and then shot at the surface of the specimen to be cleaned. Subsequently, Lie^14^ and others in the United States used dry ice foam as a solidifying core to prepare ice jets and performed experimental studies on descaling and grooving. However, because of the problems involved in the preparation, storage, and transportation of ice particles, it has not been widely promoted and applied^15–17^. Shishkin proposed a mechanical ice-breaking method. However, the broken ice particles need to be stored in a low-temperature environment for a long time, leading to higher energy consumption and easy adhesion of the ice particles^18,19^. Avoiding ice particle storage by instant preparation and utilization is the key to facilitating the widespread use of ice abrasive air jets.

Existing instantaneous preparation methods for ice particles mainly include direct refrigerant contact, vacuum rapid cooling methods and crushed ice method. New Jersey Institute of Technology's Geskin^20^ at New Jersey Institute of Technology had developed an ice crushing system and water freezing technique that can prepare ice pellets for surface cleaning at a certain rate. Based on the refrigerant direct contact method, Gieseler et al.^21^ directly sprayed liquid droplets atomized by an ultrasonic nozzle into a glass container containing refrigerant and prepared almost spherical ice particles. Li et al.^12^ used the spray generated by the atomization nozzle to make direct contact with the atomized liquid nitrogen, which rapidly cool in a low-temperature environment by phase change to form ice particles. Owing to the structure of the atomization nozzle, the ice particle size is small, with an average diameter of 100 ${\upmu}$m, and is not adjustable. The temperature distribution in the chamber is non-uniform and uncontrollable, resulting in irregular ice particle hardness. The ice particles accumulate at the bottom of the ice chamber and are heavily bonded to each other, blocking the outlet and resulting in low ice particle utilization. Abrasive particle size, hardness and mass flow rate have a certain effect on the jet erosion effect^2,22,24^. Shin et al.^23^ used the flash principle of liquid drops in a vacuum environment to form tiny ice particles and designed a tiny and fast ice-particle-making system, which consisted of a water supply device, vacuum chamber, steam compressor, steam condenser, and vacuum pump. Spherical ice particles with a diameter of 50 μm and temperature of 0 °C were produced continuously using this device. However, this method requires too many equipment and is a complex process with high energy consumption, low ice-making efficiency, and no control over ice particle size and temperature. Liu et al.^24^ found that abrasive hardness is the main factor affecting the erosion effect. Abrasive hardness is quadratically related to erosion depth, and if the particle size is too small or too large, it cannot effectively change the erosion effect. Particle size and hardness are the key factors affecting the erosion efficiency, as the size and hardness of ice particles are the key factors that affect the efficiency of ice abrasive air jet surface treatment^25,26^.

Therefore, in the instant preparation process of ice particles, effective control of the particle size and hardness must be achieved. Energy consumption can be reduced by improving the ice particle preparation efficiency, increasing the heat utilization rate, and shortening the freezing time. In this study, we propose a new method for the preparation of ice particles, investigate the phase change heat transfer mechanism of droplets in a low-temperature environment. Castillo^27^ had measured the freezing process of droplets with an infrared camera and numerically simulated the transient solidification process of droplets on cooled substrates with ANSYS Fluent, and the two results achieved a high degree of agreement. Similarly, Bodaghkhani^28^ investigated the freezing process of droplets on various wet-coolable surfaces by means of experimental and numerical simulations. Therefore, in this paper, we investigate the freezing process of liquid droplets from a combination of simulation and experiment, and compare and analyze the ice-forming mechanism of liquid droplets under different low-temperature conditions, and find a method with low energy consumption for instant preparation of ice particles. In addition, we determine the key factors that affect the formation of ice droplets, analyze the changing principle of the droplets between the key factors, and provide a reference for the instant preparation of ice particles.

## Ice particles instant preparation method

### Preparation principle

**Figure 1:**
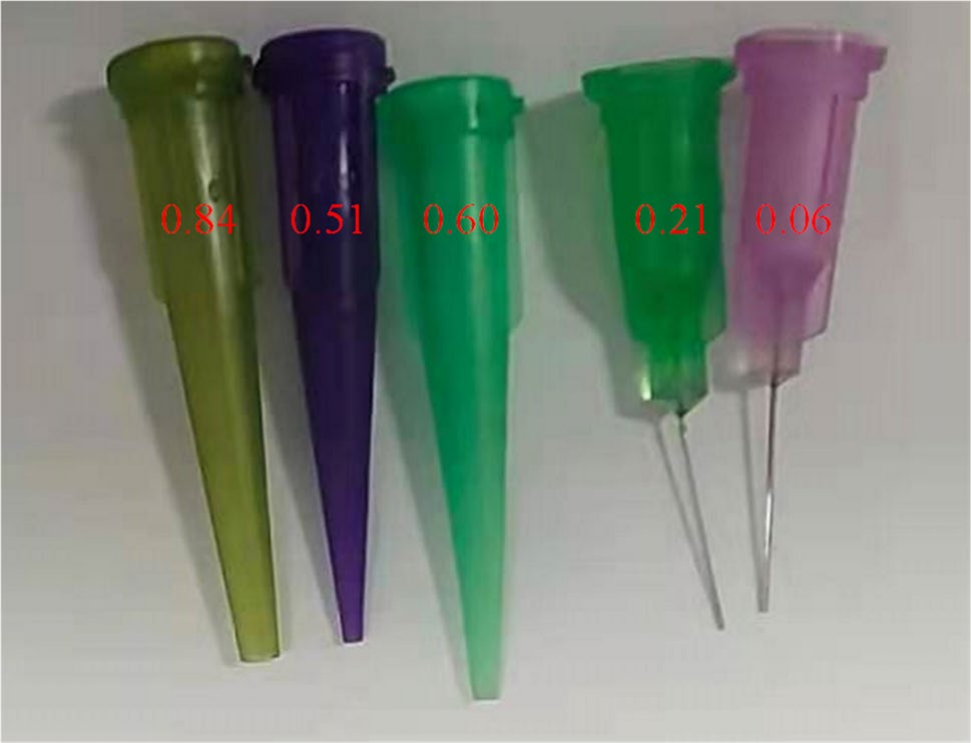
Specification of droplet generator outlet.

The freezing principle of heterogeneous nucleation, based on the notion that droplets exothermically phase change on a low-temperature substrate to form ice particles^29–31^, is used to shorten the freezing time of droplets and improve the thermal conductivity of droplet heat transfer^32,33^. The substrate temperature is controlled by the liquid nitrogen flow rate, the substrate temperature is controlled by the solenoid flow meter and temperature sensor linkage control of liquid nitrogen flow rate, the error range of the solenoid flow meter is ± 0.5%, measurement range is 1.19–119.4 m^3^/h; the water source in the water tank is used as the raw material for the droplet generation device, the droplet particle size is controlled by the pneumatic ball valve as well as the droplet outlet diameter together, where the pneumatic ball valve supply pressure range is 0.4–0.8 Mpa, measurement range is 1–4 Mpa, the error range ± 0.2%, the droplet generator is shown in Fig. 1, and its specifications and droplet generation dimensions are shown in Table 1. Meanwhile, the initial temperature of ice particles can be controlled by pre-cooling. The temperature of the ice particles is monitored by a thermocouple, and the hardness of the ice particles is controlled by its temperature^34^, after the ice particles on the substrate have completely cooled, the handle controlled the scraper to move up and down along the wall to scrape off the ice particles to the collection device. The high-speed airflow accelerated the ice particles, thus achieving uniform utilization and avoiding bonding and storage in the production process. The physical parameters of the substrate used in this experiment are listed in Table 2. The ρ, λ and ${C}_{p}$ in the table represent the density, thermal conductivity and constant pressure specific heat of the material used to make the ice cavity substrate, respectively.

**Table 1:** Droplet generator specifications.

Outlet diameter (mm)	Droplet radius ($\text{mm})$
0.84	2.526
0.60	2.013
0.51	1.542
0.21	1.063
0.06	0.563

**Table 2:** Physical parameters of the substrate.

$\rho$ ($\text{kg}/{\text{m}}^{3}$)	$\lambda$ ($\text{W/(m}\cdot \text{k)}$)	${C}_{p}$ ($\text{j/(kg}\cdot \text{k)}$)
8030	16.27	502.48

### Experimental equipment

**Figure 2:**
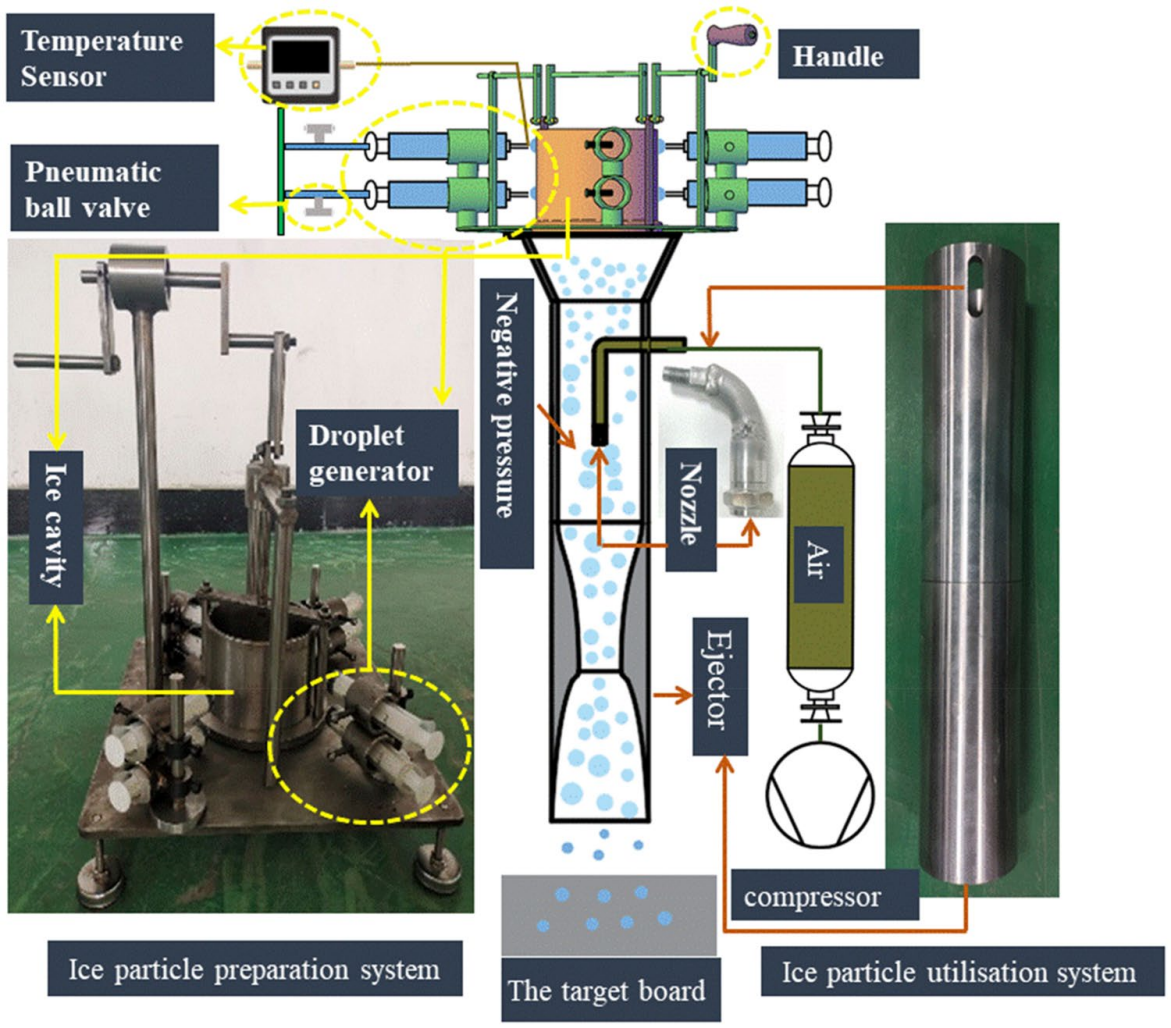
Experimental system for ice abrasive air-jet.

The experimental system of the ice abrasive air jet is shown in Fig. 2. The experimental system included an instant ice particle preparation system and an ice particle utilization system. This paper described a system for the instantaneous preparation of ice particles, comprising a droplet generation device, an ice-forming device, and a data logging device. The ice-forming unit consisted of an ice cavity, a liquid nitrogen tank, and a scraper; the data logging unit consisted of a data logger and multiple temperature sensors. The temperature sensors used two types of T-type omega thermocouples with temperature ranges of 73–373 $\text{K}$ and 173–533 $\text{K}$. The thermocouple beads head diameters were 1.6 $\text{mm}$ and 0.254 $\text{mm}$ respectively, and all thermocouples were sampled at 10 Hz using a data logger, with an error range of ± 0.5 °C, all of which can meet the requirements for droplet freezing time and maintain the temperature inside the ice-forming cavity surface to determine the surface temperature of the droplet. The ice particle ejection system consisted of an ejector, nozzle, compressor, cylinder, pressure-reducing valve, and collection device. As the experimental system diagram in Fig. 2, when the experimental system is operating, the solenoid valve flow meter and temperature sensor linkage control the flow of liquid nitrogen, use the water source in the tank as the raw material for the droplet generation device to avoid the uneven droplet experience due to the uneven water pressure, and control the droplet particle size jointly by the valve with air pressure and the droplet outlet diameter in the generator. The spindle control scraper will be made of ice particles scraped down to the collection device, ice particles through the high-speed air curl sucked into the lead pipe, and then through the nozzle and then sprayed out to the surface of the workpiece for cleaning operations.

## Numerical simulation

### Control equations

The Solidification melting model method was proposed by Voller and Prakash et al. and is used in FLUENT as a porous enthalpy method in the solidification melting process^35^ The phase change interface is represented by the liquid phase coefficient, and the total enthalpy $H$ of the droplet is determined by the thermodynamic enthalpy $h$ and latent heat $\Delta H$ of the mixture at a certain fluid volume fraction, and the thermodynamic equation for solidification melting.

1 $$H=h+\Delta H,$$

2 $$h={h}_{ref}+{\int }_{{T}_{ref}}^{T}{c}_{p}dT,$$

where ${h}_{ref}$ is the reference enthalpy, ${T}_{ref}$ is the reference temperature, ${C}_{p}$ is the specific heat at a constant pressure. The latent heat of the mixture under a certain fluid volume fraction is

3 $$\Delta H=\beta L,$$

where $L$ is the latent heat of solidification, and $\beta$ is the volume fraction of the fluid and defined as^36^:

when $$T<{T}_{s}$$

$$\beta =0$$

when $$T>{T}_{l}$$

$$\beta =1$$

when $${T}_{s}<T<{T}_{l}$$

$$\beta =\frac{T-{T}_{s}}{{T}_{l}-{T}_{s}},$$

where ${T}_{s}$ is the solidification temperature, ${T}_{l}$ is the melting temperature, and the difference between ${T}_{l}$ and ${T}_{s}$ is the phase transition temperature zone. The energy control equation for solidification and melting in FLUENT is

4 $$\frac{\partial }{\partial t}\left(\rho H\right)+\nabla \cdot \left(\rho \overrightarrow{V}H\right)=\nabla \cdot \left(\kappa \nabla T\right)+S,$$

where $\rho$ is the density, $\overrightarrow{V}$ is the fluid velocity, and $S$ is the source term. Of the multiphase flow models in FLUENT, only the VOF model can be used for solidification melting^32,33^ the method was proposed by Hirt and Nichols^37^. The VOF model is used to simulate the flow of two or more immiscible fluids by solving separate momentum equations and treating the volume fraction of each fluid passing through the region. The two interfaces are traced by calculating the volume fraction, ${C}_{K}$, occupied by the kth phase in each grid.

5 $${C}_{k}\left(x,y,z,t\right)=\left\{\begin{array}{c}1\\ 0\\ 0-1.\end{array}\right.$$

In any grid,

6 $${\sum }_{1}^{n}{C}_{k}=1.$$

The continuous equation is solved as

7 $$\frac{\partial }{\partial \tau }\left({C}_{k}{\rho }_{k}\right)+\nabla \left({C}_{k}{\rho }_{k}{u}_{k}\right)=0.$$

Each fluid phase is governed by the same mass conservation, momentum conservation, and energy conservation equations^38^.

8 $$\frac{\partial \rho }{\partial \pi }+\frac{\partial }{\partial {x}_{i}}\left(\rho {u}_{i}\right)={S}_{m},$$

9 $$\frac{\partial }{\partial \tau }\left(\rho {u}_{i}\right)+\frac{\partial }{\partial {x}_{i}}\left(\rho {u}_{i}{u}_{j}\right)=-\frac{\partial p}{\partial {x}_{i}}+\frac{\partial {\tau }_{ij}}{\partial {x}_{j}}+\rho {g}_{i}+F,$$

10 $$\rho \frac{\partial H}{\partial \tau }=\nabla \left(\kappa \nabla t\right).$$

The source term in the momentum equation is mainly reflected in the role of surface tension.

11 $$F=\sigma {K}_{\kappa }\frac{\rho \nabla {C}_{k}}{1/2({\rho }_{g}+{\rho }_{l})} .$$

When the solidification and melting model in FLUENT is coupled with the VOF model in multiphase flow, the algorithm first calculates the different fluid substance distributions and then the solid–liquid phase distribution of the substance. Thus, the evolution of the solid–liquid phase distribution of the solidification process inside the droplet is obtained.

12 $$\frac{\partial }{\partial \tau }\left(\rho {u}_{i}\right)+\frac{\partial }{\partial {x}_{j}}\left(\rho {u}_{i}{u}_{j}\right)=-\frac{\partial P}{\partial {x}_{i}}+\frac{\partial {\tau }_{ij}}{\partial {x}_{j}}+\rho {g}_{i}+F+f+{S}_{u},$$

where $\rho$ represents the liquid density, $P$ is the pressure, $f$ is the viscous acceleration, $F$ is the surface tension term, $g$ is the gravitational acceleration, and $S$ is the source term that changes with temperature in the solidification model. When the material is completely in liquid state with $S=0$, substance solidification in full will be of great value; after referring to the relevant literature, it was set to 100,000^39,40^.

### Physical model and meshing

**Figure 3:**
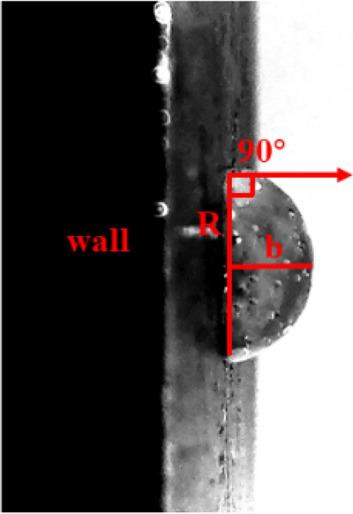
Droplet contact angle and height.

**Figure 4:**
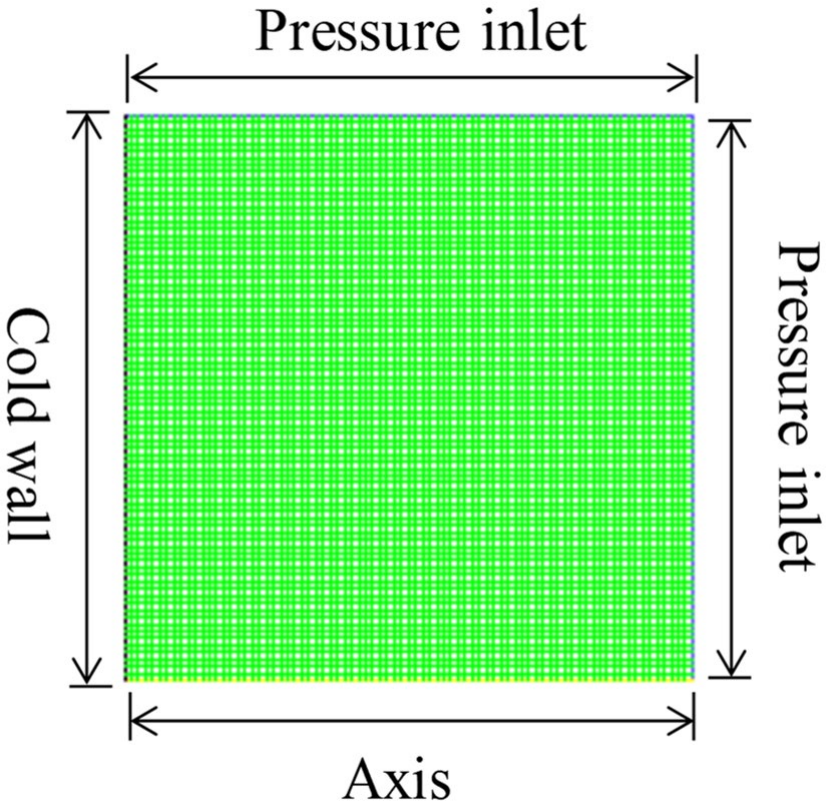
Calculation grid.

**Figure 5:**
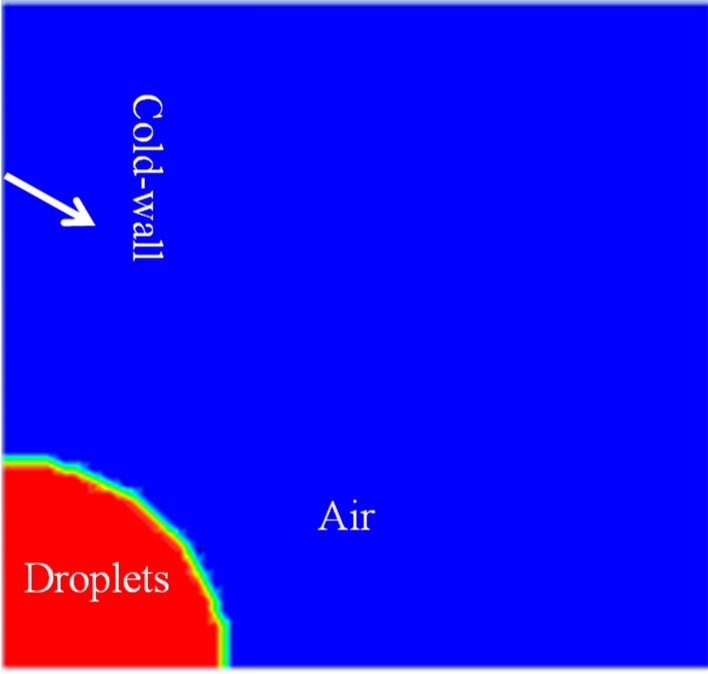
Initial phase.

In order to clarify the contact state of the droplet with the vertical substrate, a droplet generator was used to generate the droplet on the substrate. As shown in Fig. 3, the droplet height and contact angle were measured. The average height and contact angle were derived by repeating the experiment several times. The physical model of the numerical simulation was identical to the droplet morphology. The space where the droplets are located is a three-dimensional open space, and the numerical simulation is simplified to a two-dimensional central axisymmetric plane, with the computational domain divided into a low-temperature wall surface and an open space. ICEM CFD was used for structured meshing, and the entire area consisted of 6724 meshes, with quality above 0.9. The calculation area, mesh division, and initial state phase diagram are shown in Figs. 4 and 5.

Strub used a syringe to inject droplets to simulate the cooling crystallization process in which the droplets have a complete spherical shape, while Tabakov simulated the crystallization process of droplets on the wall in which the droplets have a truncated spherical shape. The droplet exiting the droplet generator is a complete sphere, unlike the droplet on the wall^41,42^. As shown in Table 3, the contact angle obtained from the repeated experiments was used to set the basic parameter specifications for the droplet on the wall and the standard deviation of contact angle is about 0.18–0.21, and the standard deviation of droplet average height is about 0.005–0.0052.

**Table 3:** Specifications for basic droplet parameters.

$r$ ($\text{mm}$)	0.5	1	1.5	2	2.5
$R$ ($\text{mm}$)	0.630	1.260	1.890	2.520	3.150
$b$ ($\text{mm}$)	0.630	1.260	1.890	2.520	3.150

### Boundary conditions and initial parameters

The boundary conditions in the calculation area are shown in Fig. 4, with the bottom edge set as the central axis of symmetry, the right and top edges are the pressure inlet boundary conditions, the left side is the cold wall surface. The adiabatic boundary conditions and the non-slip adiabatic wall surface boundary are set simultaneously. The acceleration due to gravity along the Y-axis was set to − 9.8 $\text{m}/{\text{s}}^{2}$, the solidification and melting temperatures of the droplet were set to 273 $\text{K}$ in this simulation, the latent heat of solidification was set to 333,146 $\text{J}$, the surface tension of the droplet was 0.073 $\text{N}/\text{m}$, the thermal conductivity of the droplet, and the specific heat of constant pressure varies with temperature. The thermophysical properties of the liquid droplet, solid ice particles, and air during the simulation are shown in Table 4:

**Table 4:** Physical properties of droplets and air.

Material	${C}_{p}$ ($\text{j/(kg}\cdot \text{k)}$) (specific heat capacity)	$\rho$ ($\text{kg}/{\text{m}}^{3}$) (density)	$\lambda$ ($\text{W/(m}\cdot \text{k)}$) (thermal conductivity)	$\mu$ ($\text{Pa}\cdot \text{s}$) (dynamic viscosity)
Liquid phase	${C}_{p1}$	999.9	${\lambda }_{1}$	0.001003
Solid phase	${C}_{p2}$	920	${\lambda }_{2}$	0.001003
Air	1005.9	1.4	0.0242	1.7894e−05

When the temperature of the droplet is $T\in (273\text{ K }, 398\text{ K })$,

13 $${C}_{p1}=4795.45455-2.1363T,$$

14 $${\lambda }_{1}=0.07685+0.0023T.$$

When the temperature of the droplet is $T\in \left(253\text{ K }, 273\text{ K }\right),$

15 $${\lambda }_{2}=4.19055-0.00709T,$$

16 $${C}_{p2}=-12.397+7.789T.$$

### Numerical simulation program

The model in this paper is a low-velocity incompressible flow problem, so the pressure-based computational solver, i.e., the discrete solver, is chosen. For the kinematic phase interface in the multiphase flow problem, the PISO method can better satisfy the momentum equation and continuity equation. The calculation type is transient simulation, and the time step is set to 0.00001 s according to the calculation requirements, and the number of iterations for each time step is 20 to ensure that the results converge within each step, and the computational time domain is adjusted according to the solidification time of droplets under different environmental conditions. In the instant preparation method of ice particles used in this study, the wall temperature, initial droplet temperatures, and droplet radius are the key factors affecting droplet freezing time. Their effects on the droplet ice-forming principle were analyzed using the single variable method, taking the droplet initial temperature as an example, fixing the wall temperature as 173 K, droplet radius as 1 mm, and then altering the droplet initial temperature in turn to analyze the effect of droplet initial temperature on ice-forming. As with the other two approaches, a total of 15 simulation scenarios were designed according to the instant preparation method. The simulation scenarios are shown in Table 5.

**Table 5:** Single factor simulation data table.

${T}_{0}$ ($\text{K}$)	${T}_{wall}$ ($\text{K}$)	$r$ (mm)
278	173	1
283	173	1
288	173	1
293	173	1
298	173	1
288	173	0.5
288	173	1
288	173	1.5
288	173	2
288	173	2.5
288	153	1
288	163	1
288	173	1
288	183	1
288	193	1

## Results and discussion

### Droplet to ice-forming process

The phase transition heat of droplets with different radii, initial temperatures, and wall temperatures was investigated to obtain the solidification of droplets on low-temperature walls, taking into account both the variation of physical parameters such as thermal conductivity and specific heat capacity with temperature, calculated using the simulation conditions in Table 5.

**Figure 6:**
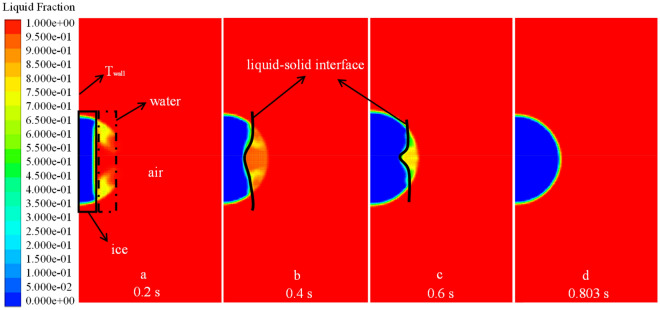
*r* = 1 $\text{mm}$ liquid–solid phase volume fraction.

**Figure 7:**
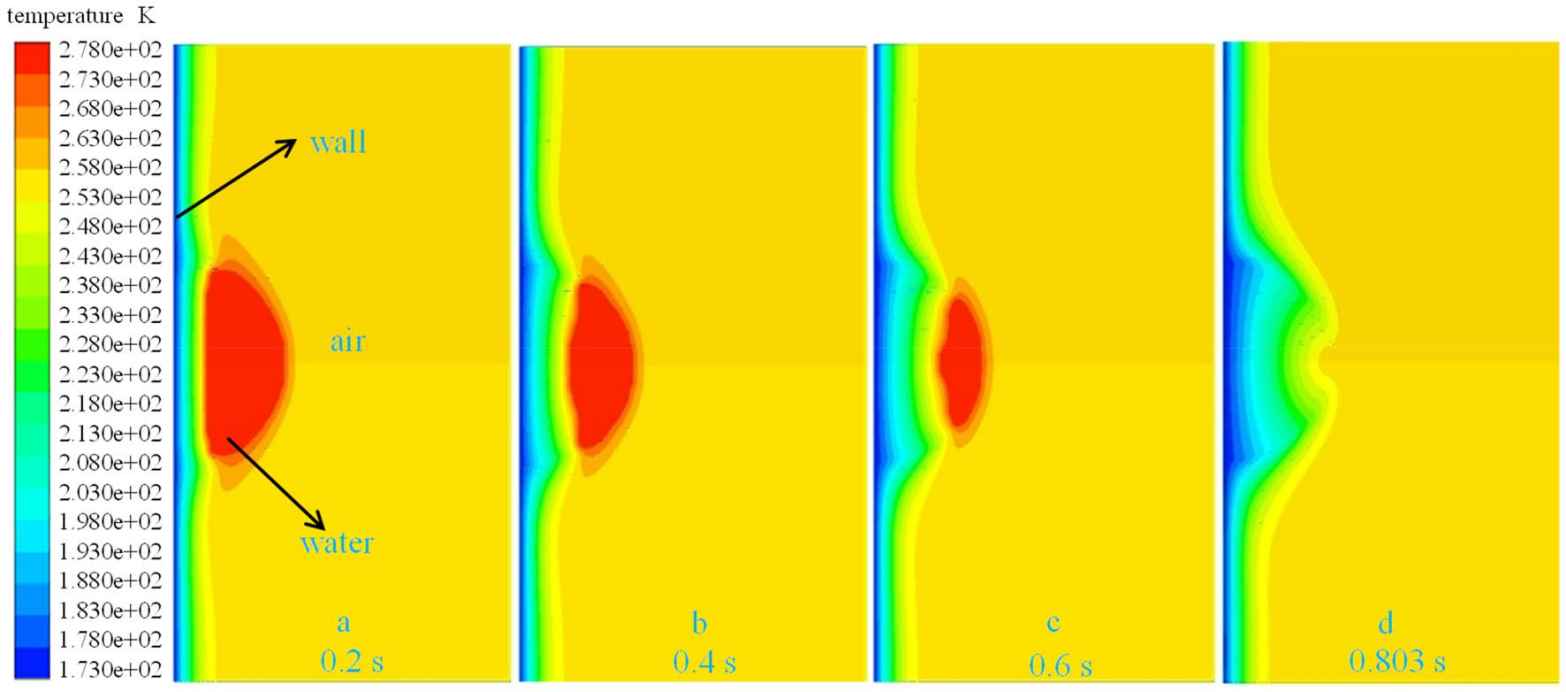
*r* = 0.5 $$\text{mm}$$ temperature vector.

As an example, a droplet of radius 1 $\text{mm}$ with an initial temperature of 278 $\text{K}$ and a wall temperature of 173 $\text{K}$ was used to illustrate a droplet’s thermodynamic characteristics during ice-forming. The volume fraction of the liquid–solid phase and the temperature vector diagram of the droplet at different moments reflect its ice-forming state. As shown in Figs. 6 and 7, the droplet in contact with the wall first undergoes a phase change and continues to expand outward in the radial direction of the droplet, as the volume fraction of the solid phase increases and the temperature of the droplet decreases. Until 0.2 $\text{s}$, the same principle of change was maintained, the solid phase volume fraction of the droplet increased uniformly, the liquid–solid phase dividing line increased linearly, and the droplet cooling process occurred uniformly. When the phase change proceeds to $r=0.32 b$, the liquid–solid phase boundary no longer remained straight. The phase change at the center of the droplet became slower, whereas the phase change at the boundary was relatively fast, so the temperature dividing line of the droplet and the liquid–solid phase dividing line showed a parabolic shape. As shown in Figs. 6, 9 and 10, the parabolic-like parabolas for different radius conditions have the same pattern, that is, the concavity of the parabolic-like line increases as the phase transition time advances, exactly as shown in Figs. 6d, 9d and 10d.

**Figure 8:**
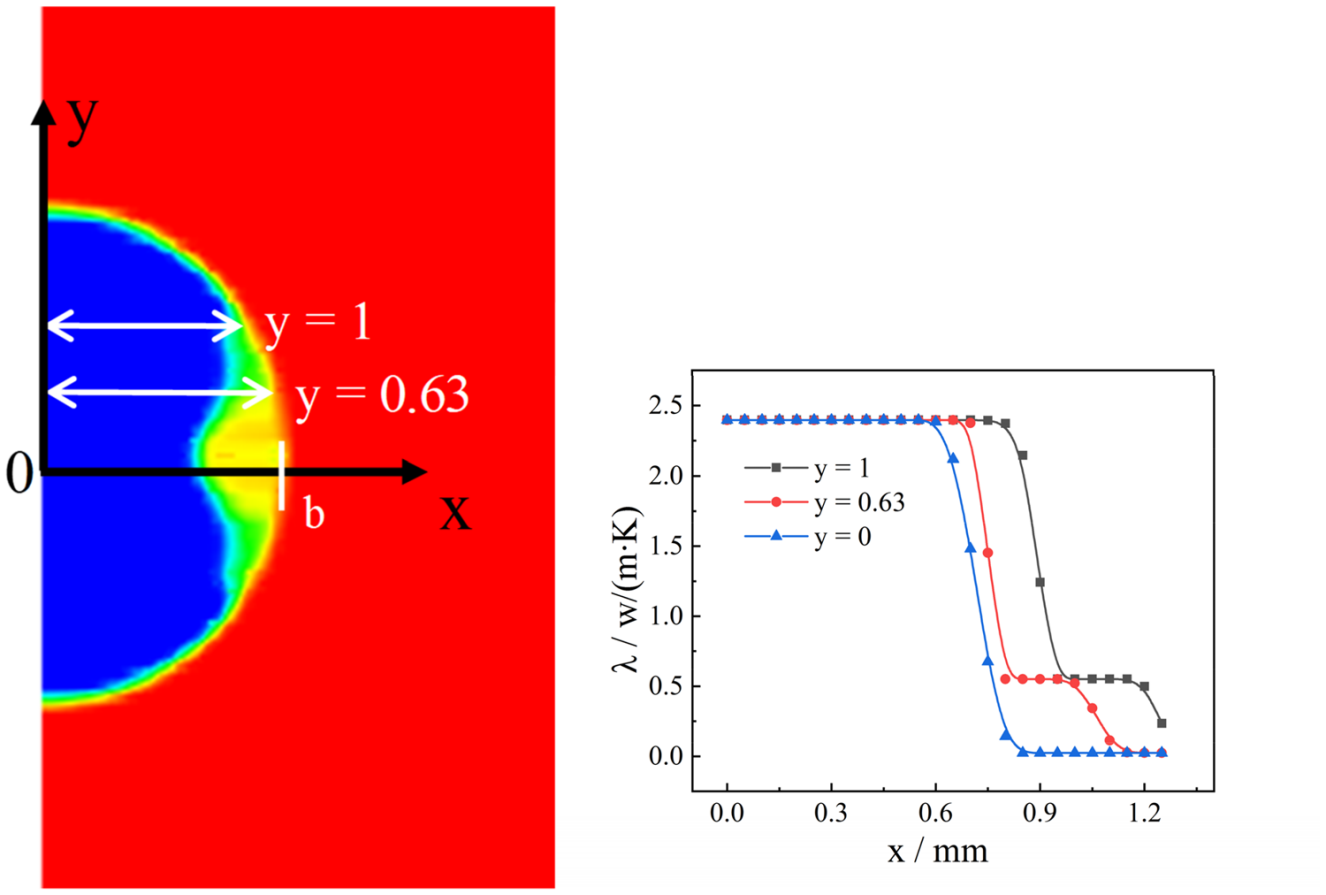
Radial thermal conductivity at 0.6 s for 1 $\text{mm}$ droplets.

**Figure 9:**
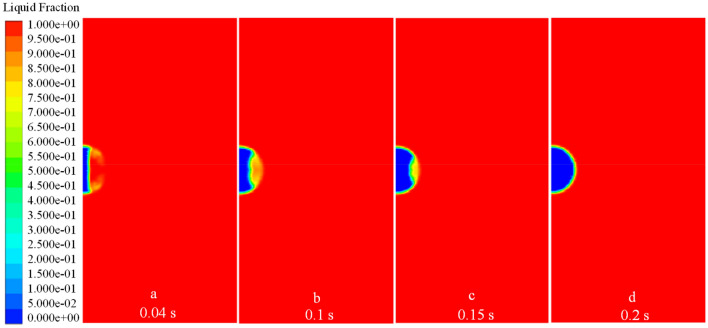
*r* = 0.5 $\text{mm}$ liquid–solid phase volume fraction.

**Figure 10:**
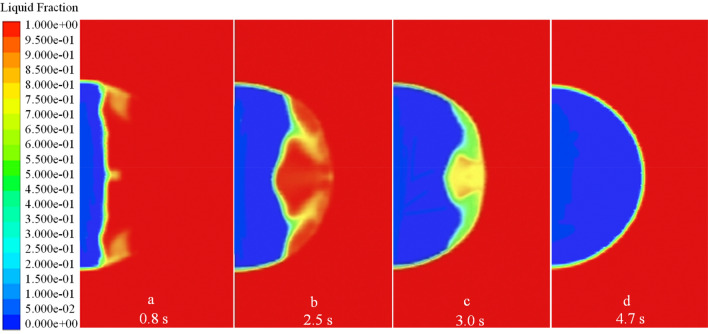
*r* = 2.5 $\text{mm}$ liquid–solid phase volume fraction.

To clarify the parabolic-like phenomenon generated at the liquid–solid phase interface, the thermal conductivity of droplets at different radial locations was extracted, as shown in Fig. 8. For example, the thermal conductivity of the droplet of radius 1 mm at 0.6 is always greater at *y* = 1. The thermal conductivity of the droplet at the center is always smaller than thermal conductivity at the ends, the reason for this phenomenon is that thermal conductivity increases after phase change, and the volume fraction of the solid phase at the droplet's boundary is larger than that at its center. Thus, the heat transfer process is accelerated, so that the change of the liquid phase at the boundary occurs first. After the phase change, the thermal conductivity continues to increase, and the cycle continues, resulting in a relatively slow heat exchange process at the center of the droplet and a slow increase in the volume fraction of the solid phase. This dividing line principle remains constant until the droplet has completed its phase change, as shown in Fig. 6c,d. This paraboloidal liquid–solid phase boundary due to thermal conductivity shows a more pronounced change with an increase in droplet size, compared to Figs. 6b,c, 9b,c, 10b,c. The liquid–solid boundary becomes more pronounced with an increase in droplet radius and greater paraboloidal concavity; hence, the temperature at the highest H-point reached a lower temperature later than the rest of the droplet interior. In the study, the completion of solidification at the highest point of the droplet was considered the completion of the solidification process for the entire droplet.

### Influence of droplet size on the ice-forming process

**Figure 11:**
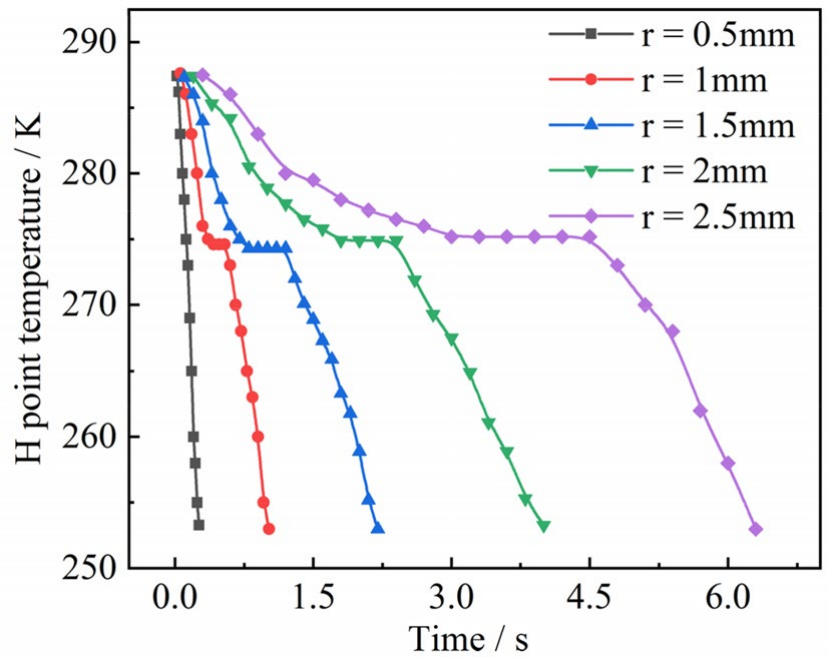
Temperature at H.

The temperature variation with time at the H-point of the droplet with a wall temperature of 173 $\text{K}$ and an initial temperature of 278 $\text{K}$ was extracted, as shown in Fig. 11. It can be seen that as the droplet radius increases, the droplet phase transition completion time and deep cooling time increase. The temperature of a droplet with a radius of 0.5 $\text{mm}$ decreased to 273 $\text{K}$ at 0.06 $\text{s}$ and 253 $\text{K}$ at 0.803 $\text{s}$. For a droplet with a radius of 2.5 $\text{mm}$, the temperature decreased to 273 $\text{K}$ at 0.85 $\text{s}$ and 253 $\text{K}$ at 5.079 $\text{s}$. The cooling rate of droplets with different radii also varies, decreasing as the radius increases. The cooling rate before and after the phase change was also different, and the cooling rate in the liquid phase was significantly lower than that in the solid phase. The droplet completed the phase change when the H-point temperature was reduced to 273 $\text{K}$, and then entered the deep cooling stage. However, the time period between the completion of the phase change and the start of cooling is not the same for droplets of different radius. When the radius is small (e.g., $r=0.5$ mm), the phase change is completed and the droplet immediately enters the deep cooling stage. When the radius increases (e.g., $r=2.5$ mm), the ice particle maintained a temperature of 273 $\text{K}$ for 3.6 $\text{s}$ before entering the deep cooling stage after the phase transition was complete. As the radius increased the longer the droplet was maintained at 273 $\text{K}$, and the slower the start of the cooling deep stage.

**Figure 12:**
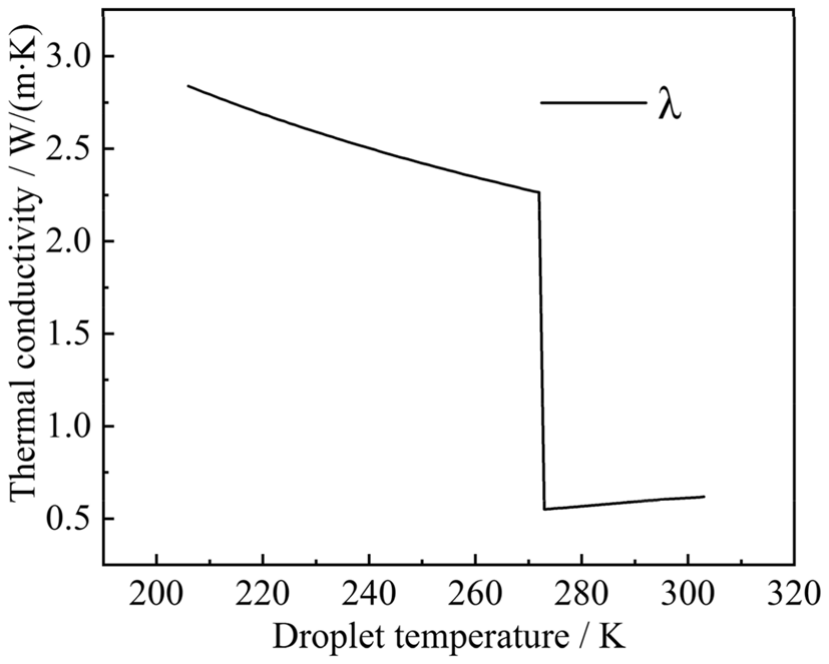
Thermal conductivity of liquid droplets.

**Figure 13:**
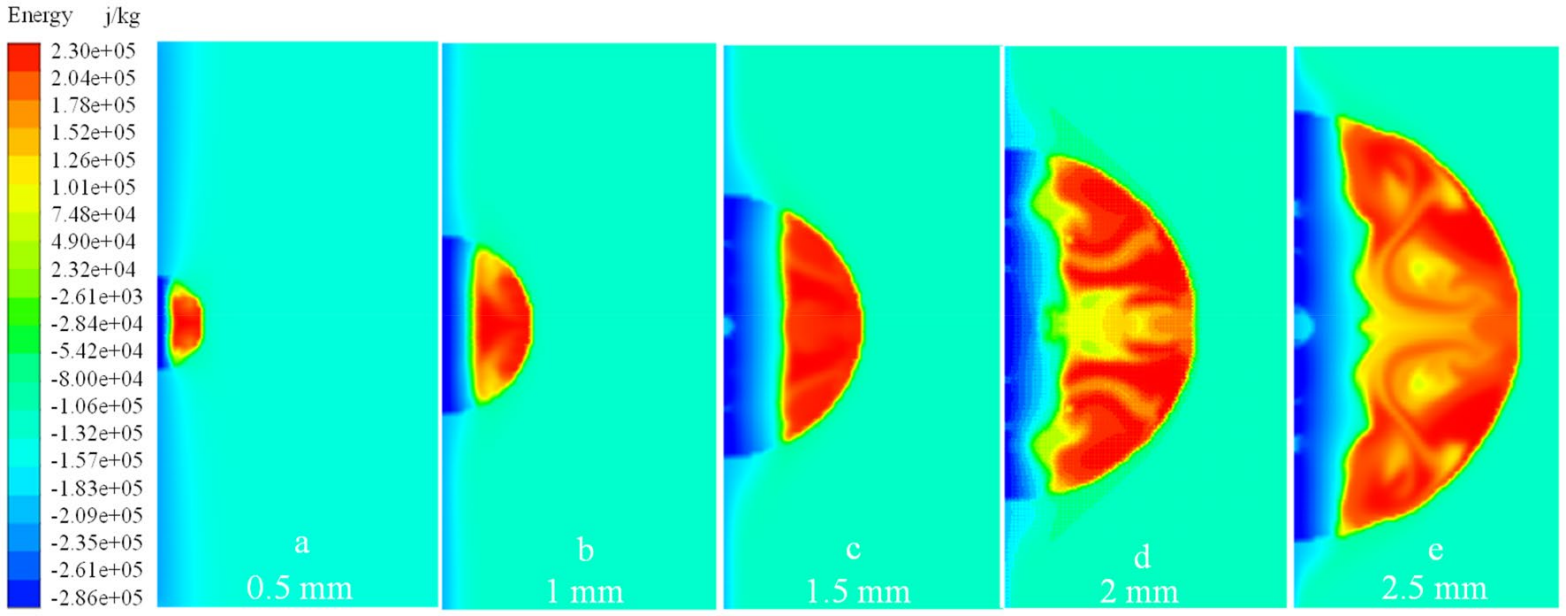
H-point for 267 $\text{K}$ liquid drop internal energy.

**Figure 14:**
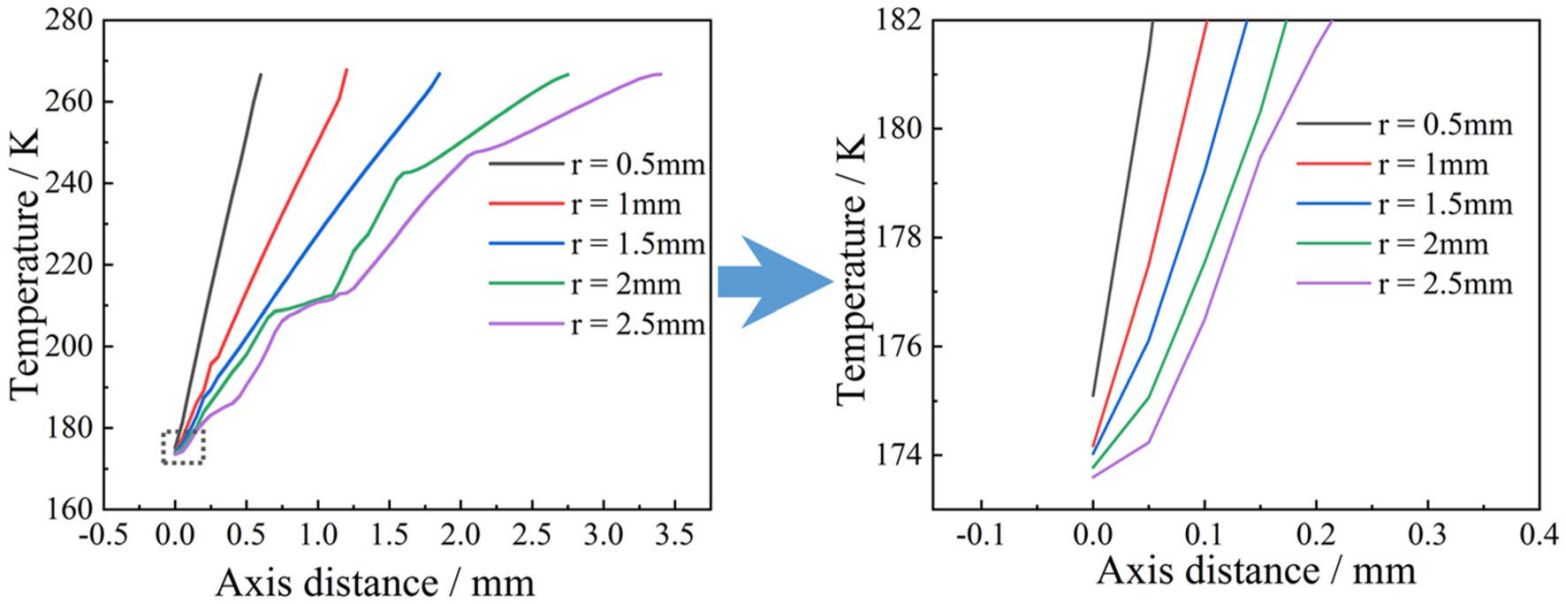
Temperature at the axis of the droplet at 267 $\text{K}$ at H.

As shown in Fig. 12, the thermal conductivity of the H-point with time was extracted to clarify the phenomenon in which the cooling rate in the deep cooling stage of the droplet is greater than the rate in the phase change stage. It can be seen that as the phase transition of the droplet continues, the thermal conductivity increases after its completion at the H-point. Moreover, as the temperature continued to decrease, the thermal conductivity continued to increase, making the cooling rate of the deep cooling stage of the droplet greater than the rate of the phase transition stage. The internal energy of droplets with different radius at the highest point of 273 $\text{K}$ was extracted, as shown in Fig. 13. As the radius of the droplet increased, the internal energy of the droplet became increase, which caused the driving energy required for the phase change of the droplet to increase, thereby increasing the droplet cooling time in the phase change stage. As shown in Fig. 14, the temperature at the central axis of the droplet at H was extracted at 267 $\text{K}$. It was found that as the droplet radius continued to increase, the temperature difference within the droplet became increasingly smaller, resulting in a slower droplet cooling rate with a larger radius.

### Influence of the initial temperature of the droplet on the ice-forming process

**Figure 15:**
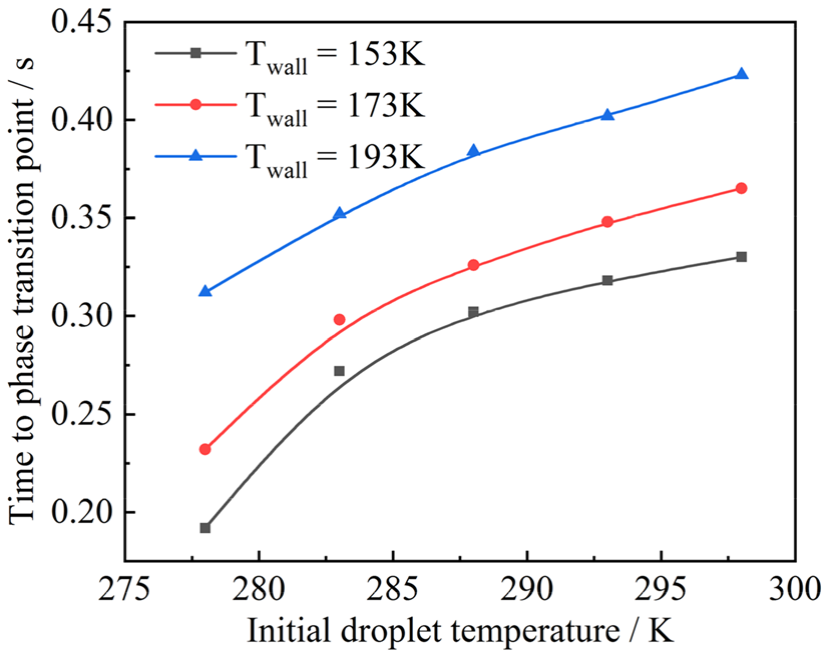
Time to phase transition point.

The effect of the initial temperature on the ice-forming process was analyzed using droplets of radius 1 $\text{mm}$. Initial wall temperatures of 153 $\text{K}$, 173 $\text{K}$ and 193 $\text{K}$ were used as an example, as shown in Fig. 15. At the same initial temperature, as the wall temperature increased, the time required for the droplet to reach the phase change point increased. As in the case of droplets at an initial temperature of 278 $\text{K}$, the phase change time of the droplet increased by 0.04 $\text{s}$ as the wall temperature increased from 153 to 173 $\text{K}$, whereas the phase change time increased by 0.08 $\text{s}$ as the wall temperature increased from 173 to 193 $\text{K}$. This trend continued as the initial temperature of the droplet increased. In addition, the time required for the droplet to reach the phase change point decreased. For example, at a wall temperature of 153 $\text{K}$, it took 0.192 $\text{s}$ for a droplet with an initial temperature of 278 $\text{K}$ to reach the phase change point and 0.33 $\text{s}$ for a droplet with an initial temperature of 298 $\text{K}$ to reach the phase change point. At a wall temperature of 193 $\text{K}$, it took 0.312 $\text{s}$ for a droplet with an initial temperature of 278 $\text{K}$ to reach the phase change point, and 0.423 $\text{s}$ for a droplet with an initial temperature of 298 $\text{K}$ to reach the phase change point. The time required to reach the phase change point varies for droplets with different initial temperatures and the same wall temperature. As the initial temperature of the droplet increased, the time required for the droplet to reach the phase change point increased, but the rate of rise gradually flattened out. For example, at a wall temperature of 153 $\text{K}$, the droplet initial temperature increases by 0.08 $\text{s}$ from 278 to 283 $\text{K}$, whereas the droplet initial temperature increases by 0.012 s from 293 to 298 $\text{K}$.

**Figure 16:**
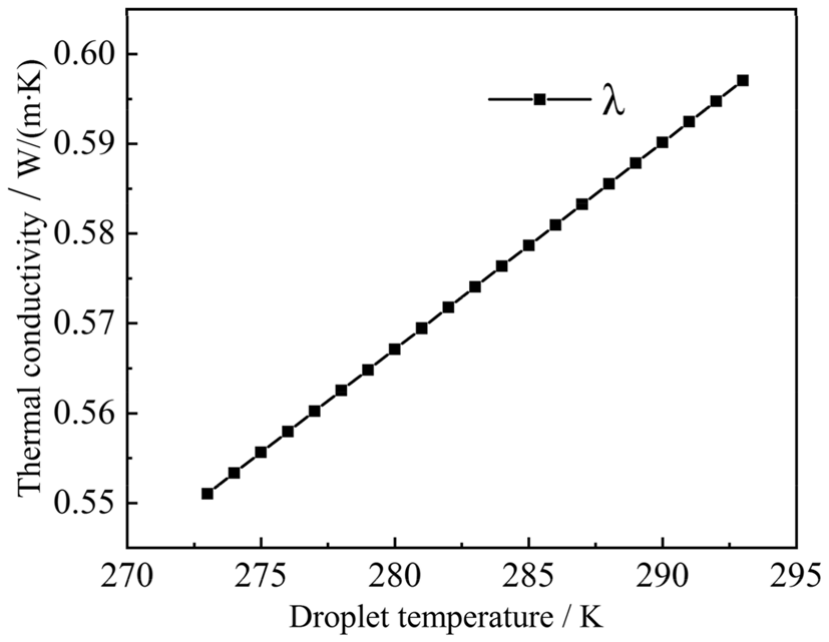
Thermal conductivity of droplets.

**Figure 17:**
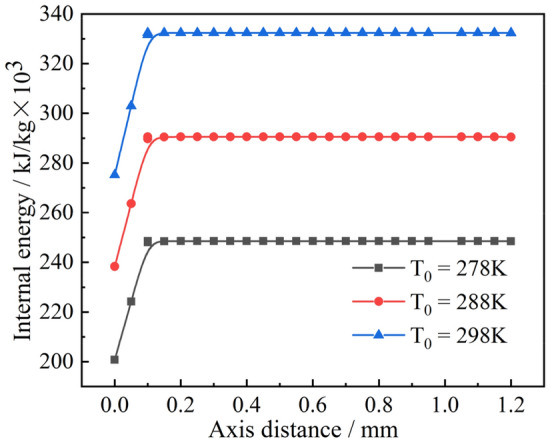
Droplet internal energy.

To further investigate the observed phenomenon, the thermal conductivity of the droplet before the phase change was analyzed, as shown in Fig. 16. The temperature difference varies depending on the droplet wall temperature. The higher the wall temperature, the smaller the temperature difference. A smaller temperature difference results in a lower thermal conductivity, as shown in Fig. 16. As time progresses, this gap continues to superimpose, resulting in an increasingly longer time to reach the phase transition. Furthermore, the internal energy of the droplets with different initial temperatures and the same wall temperature of 173 $\text{K}$ were analyzed, as shown in Fig. 17. The larger the initial temperature of the droplet, the greater its internal energy, leading to an increase in the time taken to reach the phase transition point. After the droplet reaches the phase transition point, a droplet with the same particle size and the same wall temperature undergoes the solidification process with the same variation principle, and the effect of the initial temperature of the droplet on the droplet solidification process is eliminated. As the droplet temperature increased, its thermal conductivity increased, leading to a gradual shortening of the time it took for the droplet to reach the phase change point.

### The effect of wall temperature on the ice-forming process

**Figure 18:**
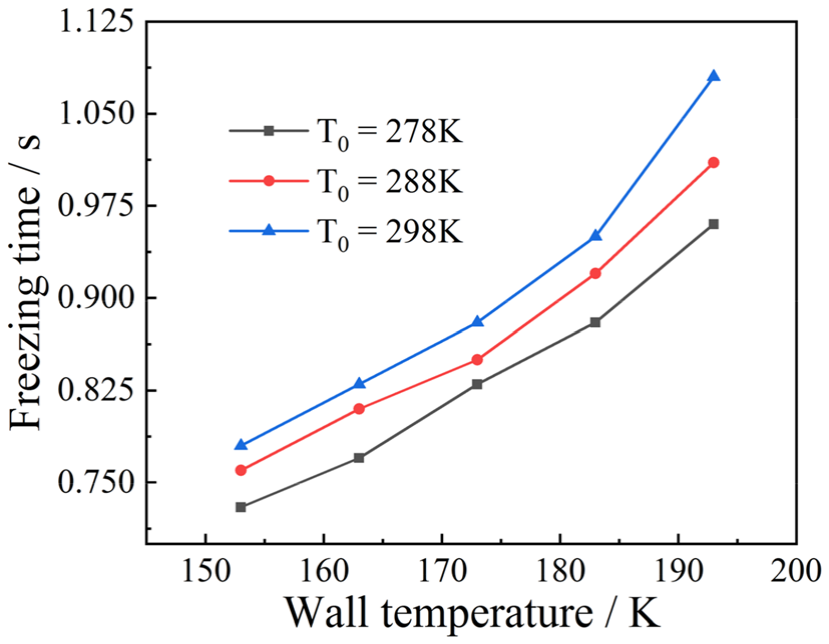
Freezing time of liquid droplets at different wall temperatures.

**Figure 19:**
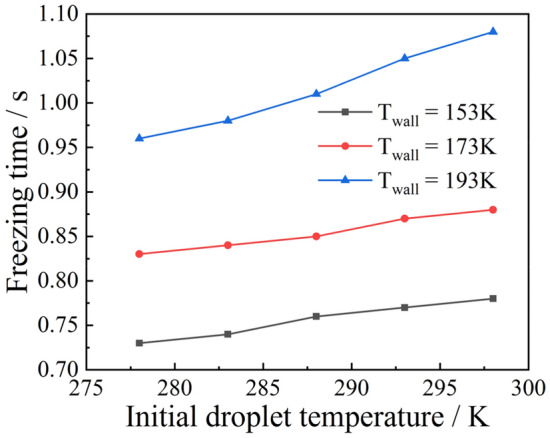
Freezing time of liquid droplets at different initial temperatures.

Using a droplet of radius 1 mm as an example, the effect of the wall temperature on the ice-forming process was analyzed, and the results of the calculation are shown in Fig. 18. It can be seen that the freezing time was proportional to the wall temperature when it was below 173 $\text{K}$. The same proportional relationship exists for different initial droplet temperatures. When the wall temperature was greater than 173 $\text{K}$, its effect on the freezing time was more prominent. This effect became more pronounced when the initial droplet temperature was high. For example, for a droplet with an initial temperature of 278 $\text{K}$, the freezing time increased by 0.08 $\text{s}$ as the wall temperature rose from 183 to 193 $\text{K}$. For a droplet with an initial temperature of 298 $\text{K}$, the freezing time increased by 0.1 s at the same wall temperature. Figure 19 shows the effect of the initial droplet temperature on the freezing time at different wall temperatures. It can be seen that under the same wall temperature conditions, the initial droplet temperature and freezing time were mostly linear, but the higher the wall temperature, the more pronounced the effect on the freezing time. In conclusion, the initial droplet temperature and wall temperature have a roughly linear relationship with the droplet freezing time, but this linear relationship decreases as the initial temperature and wall temperature increase.

**Figure 20:**
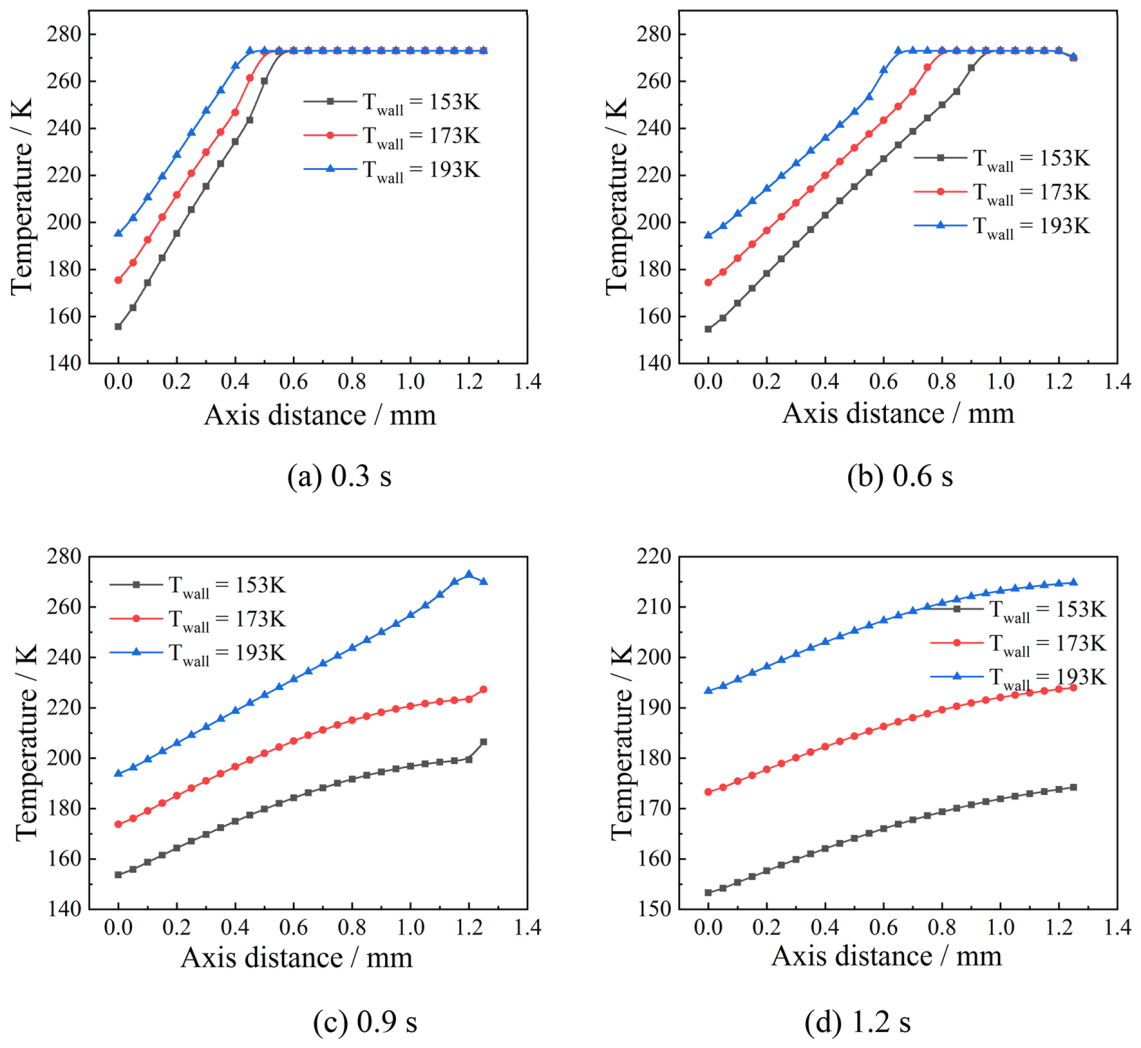
Temperature variation at the axis of the droplet.

To clarify the effect of the wall temperature on the ice-forming principle, the temperature at the axis of the droplet with an initial temperature of 278 $\text{K}$ (at the x-axis of Fig. 8) was extracted at different moments as shown in Fig. 20. It can be seen that at 0.3 s, none of the droplets at different wall temperatures completely formed ice. However, when the wall temperature was lower (153 $\text{K}$), the droplets had already formed ice at 0.55 $\text{mm}$ due to the significant temperature difference, which compared to the wall temperature of 193 $\text{K}$, the freezing thickness increased by 0.15 $\text{mm}$. As the thermal conductivity of the ice-forming part increased, the rate of icing of droplets with lower wall temperatures further increased, as shown in Fig. 20b. This trend was maintained until 0.9 $\text{s}$, when the droplet H-point at 193 $\text{K}$ was at the freezing point, that is, the end of the droplet icing phase.

In summary, the lower the wall temperature, the greater the temperature difference within the droplet, the faster the thermal conductivity, and the shorter the time it takes for the droplet to reach the phase change point. The increase in the thermal conductivity of the phase-change fraction led to a further reduction in the freezing time. The results make the disparity between the droplet ice-formation time at low and high wall temperatures increasingly large, resulting in a more pronounced effect of higher wall temperatures on the ice-formation time. The temperature gradient formed at low temperatures is an important factor affecting the rate of ice formation. A comparative analysis shows that lowering the wall temperature plays an important role in eliminating the effect of the initial droplet temperature. In this case, when the cooling time is 0.9 $\text{s}$, the initial droplet temperature has no effect on the ice-forming principle and is completely controlled by the wall temperature. This means that lowering the droplet wall temperature has a more significant effect on the droplet freezing time. When the ice particle enters the cooling deep stage, the temperature distribution and trend are the same, although the different wall temperatures give the ice pellets different final temperatures, as shown in Fig. 20d. Thus, when the temperature difference and thermal conductivity within the ice pellet are the same, different wall temperatures have no effect on the temperature distribution and trend of the ice particles.

### Ice-forming principle

The relationship between the droplet freezing time, $t \; \left(\text{s}\right),$ and the initial temperature, ${T}_{0} \; (\text{K})$, radius, $r \; \left(\text{mm}\right),$ and wall temperature, ${T}_{wall } \; \left(\text{K}\right),$ was determined by performing single-factor curve regression analysis as follows:

17 $${t}_{1}=0.9028{\text{r}}^{1.983}\left({\text{R}}^{2}=0.9998\right),$$

18 $${t}_{2}=0.0026{T}_{wall}+0.1052\left({\text{R}}^{2}=0.9826\right),$$

19 $${t}_{3}=0.0057{\text{T}}_{0}-0.1521\left({\text{R}}^{2}=0.9998\right),$$

where ${t}_{1}$, ${t}_{2}$, and ${t}_{3}$ are the relationships between each factors and icing time. To linearize it, a quantity, ${t}_{1}$, that is approximately linear with the droplet freezing time, $t$, is needed. Single-factor linear fitting was performed for the new variables after substitution. Based on the aforementioned permutation results, a multiple linear regression model of droplet freezing time was developed, as shown in Eq. (20):

20 $$t={\beta }_{0}+{\beta }_{1}{t}_{1}+{\beta }_{2}{t}_{2}+{\beta }_{3}{t}_{3}+\varepsilon ,$$

where $\varepsilon \sim (0,{\sigma }^{2})$, ${\beta }_{0}$, ${\beta }_{1}$, ${\beta }_{2},$ and ${\beta }_{3}$ are the coefficients, $\sigma$ is the standard deviation.

We obtain:

21 $$\beta =\left[\begin{array}{c}{\beta }_{0}\\ {\beta }_{1}\\ {\beta }_{2}\\ {\beta }_{3}\end{array}\right]=\left[\begin{array}{c}-2.0383\\ 1.0231\\ 2.3462\\ 0.4561\end{array}\right].$$

22 $$\begin{aligned} t & = - 2.0383 + 1.0231\left( {0.9028r^{1.983} } \right) \\ & \quad + 2.3462\left( {0.0026T_{wall} + 0.1052} \right) + 0.4561\left( {0.0057T_{0} - 0.1521} \right). \\ \end{aligned}$$

On the basis of the obtained regression equation, we performed a significance F-test on the regression model and calculated the value of F_α_ (n, m – n − 1). The number of factors n = 3 and the number of times m = 15. Taking α = 0.05, the residual sum of squares Q = 0.017616, the regression sum of squares U = 23.7378, and L_yy_ = U + Q = 23.755416, then:

$$\text{F}=\frac{U/3}{Q/11}=4940.883.$$

The F distribution table was checked and the critical value F_0.05_ (3,11) = 3.59, F ≫ F_α_. The F test results show that the regression model and regression coefficients have high significance. And the correlation coefficient test results show that the model is highly correlated with each factor.

## Experimental validation

### Experimental equipment and methods

**Figure 21:**
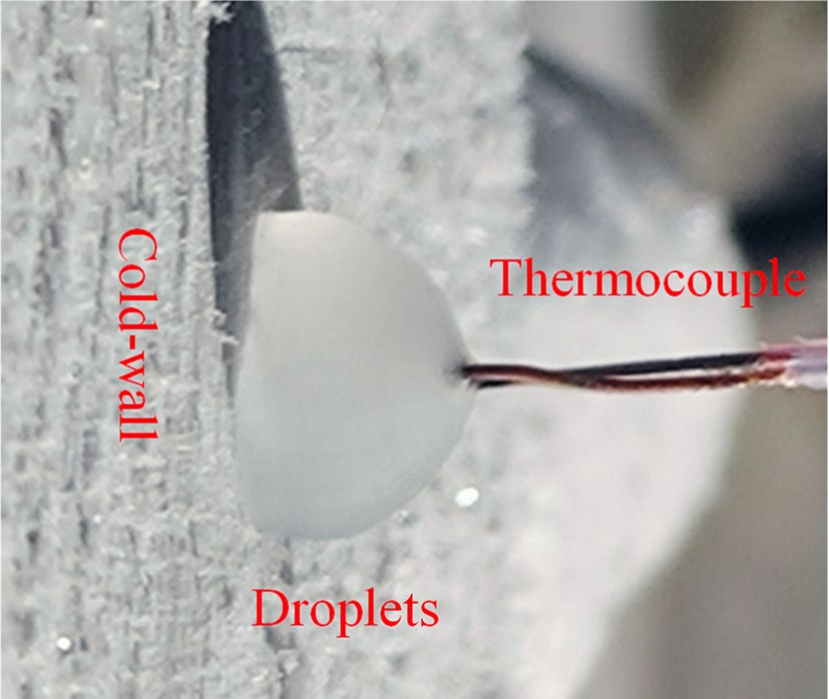
Icing of droplets.

**Figure 22:**
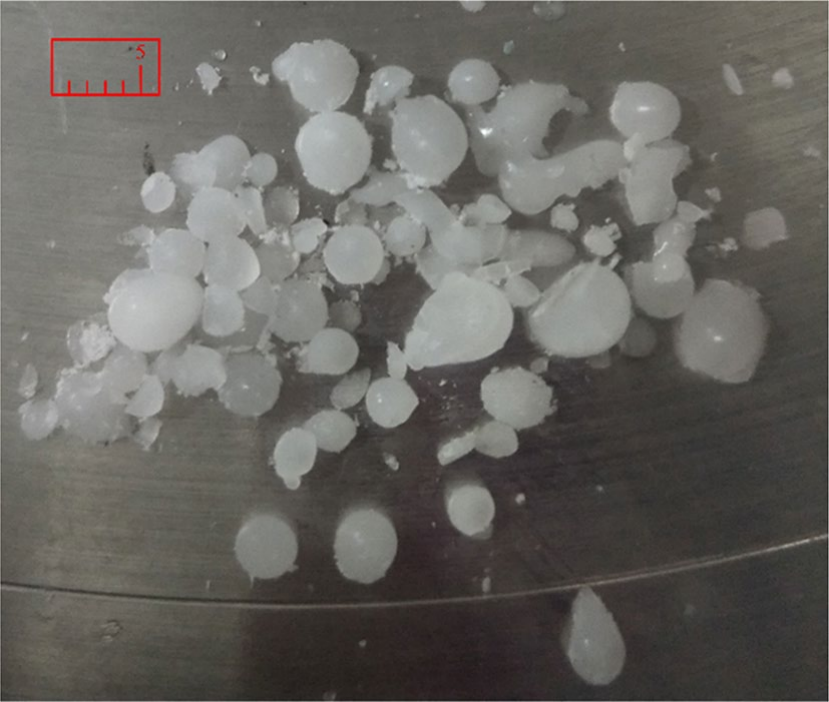
Ice particles.

The instant ice particle preparation system is shown in Fig. 2, and the thermal conductivity of the ice-forming cavity wall is the same as that in the numerical simulation. The droplet generation device was positioned perpendicular to the wall, and droplets of different initial temperatures and diameters were prepared by adjusting the pure water temperature and outlet diameter. Then, the ice cavity was rapidly cooled to a predetermined temperature by liquid nitrogen cooling. The temperature monitoring system monitors the wall surface temperature in real-time to ensure that it remains constant. To test the droplet temperature, the temperature sensor was fixed at the highest part of the droplet according to its particle size, and the droplet H-point was monitored for temperature variation under different experimental conditions, as shown in Fig. 21. Consequently, a cluster of ice particles shed from an ice-forming cavity, as shown in Fig. 22.

### Analysis of the results

**Figure 23:**
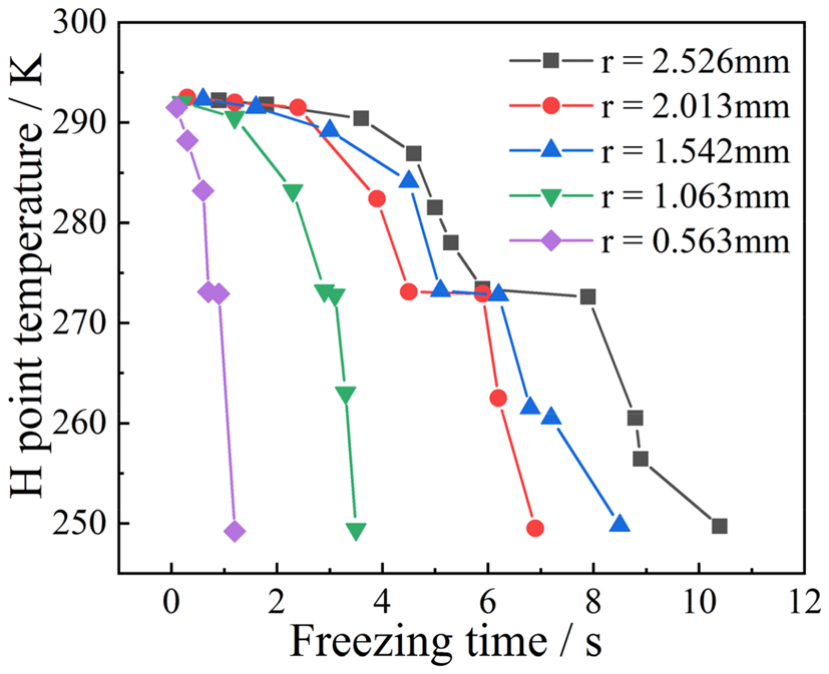
Temperature of droplet H-point.

The experimentally obtained freezing times of the droplets under different conditions are listed in Table 6. In order to verify the law of variation temperature of droplets during freezing, the droplet temperature variation at the highest point H was extracted for different particle sizes at 153 $\text{K}$ wall temperature as an example, as shown in Fig. 23. Unlike the simulation results, high droplet temperature mirrors the same principles described in “Influence of droplet size on the ice-forming process” (see Fig. 11) because impurities such as air cause the transformation point of the droplet to drop below 273 K. As shown in Fig. 23, the phase change points of droplets with different radii are different because the droplets do not contain the same amount of impurities. However, the cooling principle of droplets of different radii is consistent with the ice-forming process analyzed in “Results and discussion”.

**Table 6:** Droplet freezing time.

Data number	${T}_{0}$ ($\text{K}$)	${T}_{wall}$ ($\text{K}$)	$r$ ($\text{mm})$	$t$ (coupling value) ($\text{s}$)	$t$ (experimental value) ($\text{s}$)
1	293	153	1.542	1.26	2.8
2	293	163	1.542	1.89	3.8
3	293	173	1.542	2.017	3.1
4	293	183	1.542	2.17	3.5
5	293	153	2.013	2.52	3.9
6	293	163	2.013	3.569	5.2
7	293	173	2.013	3.6	5.5
8	293	183	2.013	4.5	5.8
9	293	193	1.542	4.9	6.2
10	293	193	2.013	5.05	6.9
11	293	153	2.526	5.2	7.0
12	293	163	2.526	5.55	7.1
13	293	173	2.526	5.89	7.2
14	293	183	2.526	6.11	7.5
15	293	193	2.526	6.6	7.8

**Figure 24:**
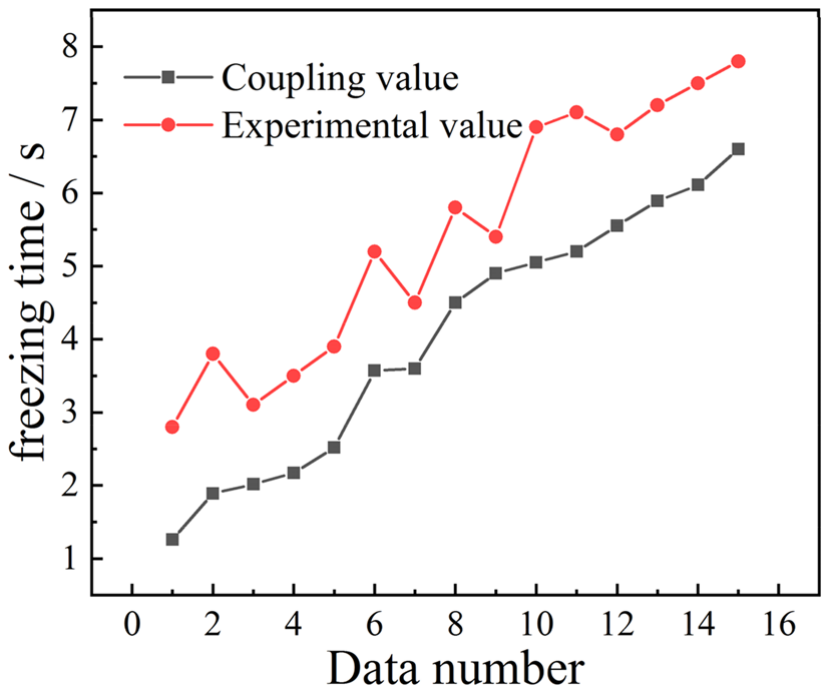
Comparison of experiment and fitting.

The fitted droplet freezing times and experimental values under the same conditions were compared to verify their accuracy, as shown in Fig. 24. The fitted freezing times showed more consistency compared to the experimental results because the droplet is in an open space, and the air temperature above the droplet does not go higher than the 253 $\text{K}$ set in the numerical simulation. In addition, human error leads to an inaccuracy in droplet size measurement, resulting in the experimental value of the freezing time being greater than the fitted result of the numerical simulation. The inconsistency in data can be overcome using the following trimming equation:

$${t}_{f}=\tau t,$$

where $\tau$ is the correction coefficient. By fitting the correction to the experimental data, the correction coefficient $\tau$ is 1.44, and the corrected droplet freezing time is

23 $${t}_{f}=1.44\left[-2.0383+1.0231\left(0.9028{r}^{1.983}\right)+2.3462\left(0.0026{T}_{wall}+0.1052\right)+0.4561(0.0057{T}_{0}-0.1521)\right].$$

## Conclusion

1. A new method for the instant preparation of ice particles on a vertical low-temperature wall is proposed using the principle of heterogeneous nucleation. Based on the experimental analysis of the droplet shape on a vertical low-temperature wall, a physical model for the numerical calculation of the droplet was established. The results show that the freezing time of the droplets is impacted by the droplet size, initial temperature, and wall temperature. Through the established droplet ice-forming model, it was concluded that the droplet ice-forming time ($<$ 12 $\text{s}$) meets the requirements of instant preparation of ice particles. In addition, the size and temperature of ice particles can be controlled, which proves the feasibility of method.
2. Based on the results of a numerical simulation study of a single droplet on a low-temperature wall, it was found that the liquid droplet close to the wall will freeze first, and the liquid–solid interface develops along the radial direction with time, but it does not increase linearly along the cross-section of the droplet. The boundary of the droplet freezes before the center of the droplet forming a parabolic liquid–solid boundary because the thermal conductivity of the boundary increases, resulting in an increase in the icing rate, and this trend continues. As the time and droplet size increased, the parabolic-like concavity increased.
3. In the freezing process, the thermal conductivity of the droplet changes as the temperature and solid volume fraction constantly change. The larger the droplet size, the longer its duration in the phase change stage. The rate in the cryogenic phase is greater than that in the phase change stage, and the droplet phase change completion time and cryogenic time continue to increase. When the initial temperature of the droplet is higher, the time for the droplet to reach the phase transition point increases gradually. The higher the wall temperature, the more noticeable the influence on the ice-forming time, and the longer the ice freezing time.

This study innovatively proposes a new method for the instant preparation of ice particles, and clarifies the relationship between droplet freezing time and droplet size, initial temperature and wall temperature in the icing process of heterogeneous nucleation, proves the feasibility of the instant preparation method, and provides new methods and ideas for the improvement of ice particle preparation methods in the future.

## Data Availability

All data included in this study are available upon request from the corresponding author.
